# Diatoms and Other Epibionts Associated with Olive Ridley (*Lepidochelys olivacea*) Sea Turtles from the Pacific Coast of Costa Rica

**DOI:** 10.1371/journal.pone.0130351

**Published:** 2015-06-17

**Authors:** Roksana Majewska, Mario Santoro, Federico Bolaños, Gerardo Chaves, Mario De Stefano

**Affiliations:** 1 Department of Environmental, Biological and Pharmaceutical Sciences and Technologies, II University of Naples, Caserta, Italy; 2 Department of Public Health and Infectious Diseases, Section of Parasitology, Sapienza University of Rome, Rome, Italy; 3 Istituto Zooprofilattico Sperimentale del Mezzogiorno, Portici, Italy; 4 Escuela de Biología, Universidad de Costa Rica, San José, Costa Rica; Scottish Association for Marine Science, UNITED KINGDOM

## Abstract

Although the sea turtles have long been familiar and even iconic to marine biologists, many aspects of their ecology remain unaddressed. The present study is the first of the epizoic diatom community covering the olive ridley turtle’s (*Lepidochelys olivacea*) carapace and the first describing diatoms living on sea turtles in general, with the primary objective of providing detailed information on turtle epibiotic associations. Samples of turtle carapace including the associated diatom biofilm and epizoic macro-fauna were collected from Ostional beach (9° 59´ 23.7´´ N 85° 41´ 52.6´´ W), Costa Rica, during the *arribada* event in October 2013. A complex diatom community was present in every sample. In total, 11 macro-faunal and 21 diatom taxa were recorded. Amongst diatoms, the most numerous were erect (*Achnanthes* spp., *Tripterion* spp.) and motile (*Haslea* sp., *Navicula* spp., *Nitzschia* spp., *Proschkinia* sp.) forms, followed by adnate *Amphora* spp., while the most common macro-faunal species was *Stomatolepas elegans* (Cirripedia). Diatom densities ranged from 8179 ± 750 to 27685 ± 4885 cells mm^-2^. Epizoic microalgae were either partly immersed or entirely encapsulated within an exopolymeric coat. The relatively low diatom species number, stable species composition and low inter-sample dissimilarities (14.4% on average) may indicate a mutualistic relationship between the epibiont and the basibiont. Dispersal of sea turtle diatoms is probably highly restricted and similar studies will help to understand both diatom diversity, evolution and biogeography, and sea turtle ecology and foraging strategies.

## Introduction

Sea turtles spend their life in the marine environment and may act as hosts to a wide variety of epibiont organisms. Their bodies (especially the broad flattened carapace) provide a very suitable substrate for periphytic growth [1, 2]. Most of these marine epibionts are unspecialized organisms (facultative commensalism) that are also often found associated with physical structures (e.g. ship hulls, dock piling, rocky surfaces), while a few are found almost exclusively on sea turtles (obligate commensalism) [2]. However, epibiosis has only recently started to receive research attention, with interest in its contribution to elucidating the cryptic life history of sea turtles, including their diet, foraging locations, migration routes and times, and stock provenance [2]. Furthermore, epibiosis may have other consequences for the host turtles, including negative effects on swimming and predatory abilities, as well as providing indications of general health status [2, 3].

Most studies to date have focused on the diversity of macro-epibiota and speculation about the possible causes and effects of their associations with host organisms [2]. Few studies have addressed macro-epibiosis from a broad community perspective [2]. Very little is known about the micro-epibiota colonizing sea turtles. Some studies mention sea turtle carapace covered with unidentified algae, but give no further information on the biotic relationships between the epibiont and the basibiont [4, 5, 6]. Little evidence is available relating the ecological and biological role of a sea turtle as a mobile substrate for the, perhaps, many microalgae that are often noted anecdotally growing epizoically on its carapace [5, 7, 8, 9].

Here, we report for the first time direct observations of the epizoic diatom community associated with the olive ridley turtle (*Lepidochelys olivacea*) carapace, providing the first detailed information on sea turtle microalgal epibionts. The study documents new and previously unknown relationships between epizoic species of diatoms and the olive ridley from the Pacific coast of Costa Rica. In addition, information on sea turtle epibiotic macrofauna that also provide a niche for microepibionts is given.

## Material and Methods

### General data

This work was a part of the international project “Communities of marine epizoic diatoms, parasites and other epibionts on sea turtles from Costa Rica: ultrastructural, taxonomic, and biogeographical analysis” led by the II University of Naples (Italy) in collaboration with the University of Costa Rica (Costa Rica) and the Sapienza University of Rome (Italy). The study was conducted in the Refugio Nacional de Vida Silvestre Ostional protected area. The study was authorized by both MINAE (Ministerio de Ambiente y Energia) and SINAC (Sistema Nacional de Áreas de Conservación) under resolution ACT-OR-DR-074-13 for the Tempisque Conservation Area (that includes Ostional). *Lepidochelys olivacea* is a protected species. In Ostional, however, these sea turtles occur in abundance, breed with success and are not currently locally endangered. A collection of epizoic diatoms, epibionts, and ectoparasites was made by scraping individual turtle carapaces with a razor. The method is not invasive, as it is limited to the most external part of the turtle carapace scutes, and it does not harm or cause the animal suffering. All sampling procedures took place as approved by MINAE under a close supervision of park rangers from SINAC. All the procedures involved respect the ethical standards in the Helsinki Declaration of 1975, as revised in 2000 and 2008, as well as the applicable national law.

**Fig 1:**
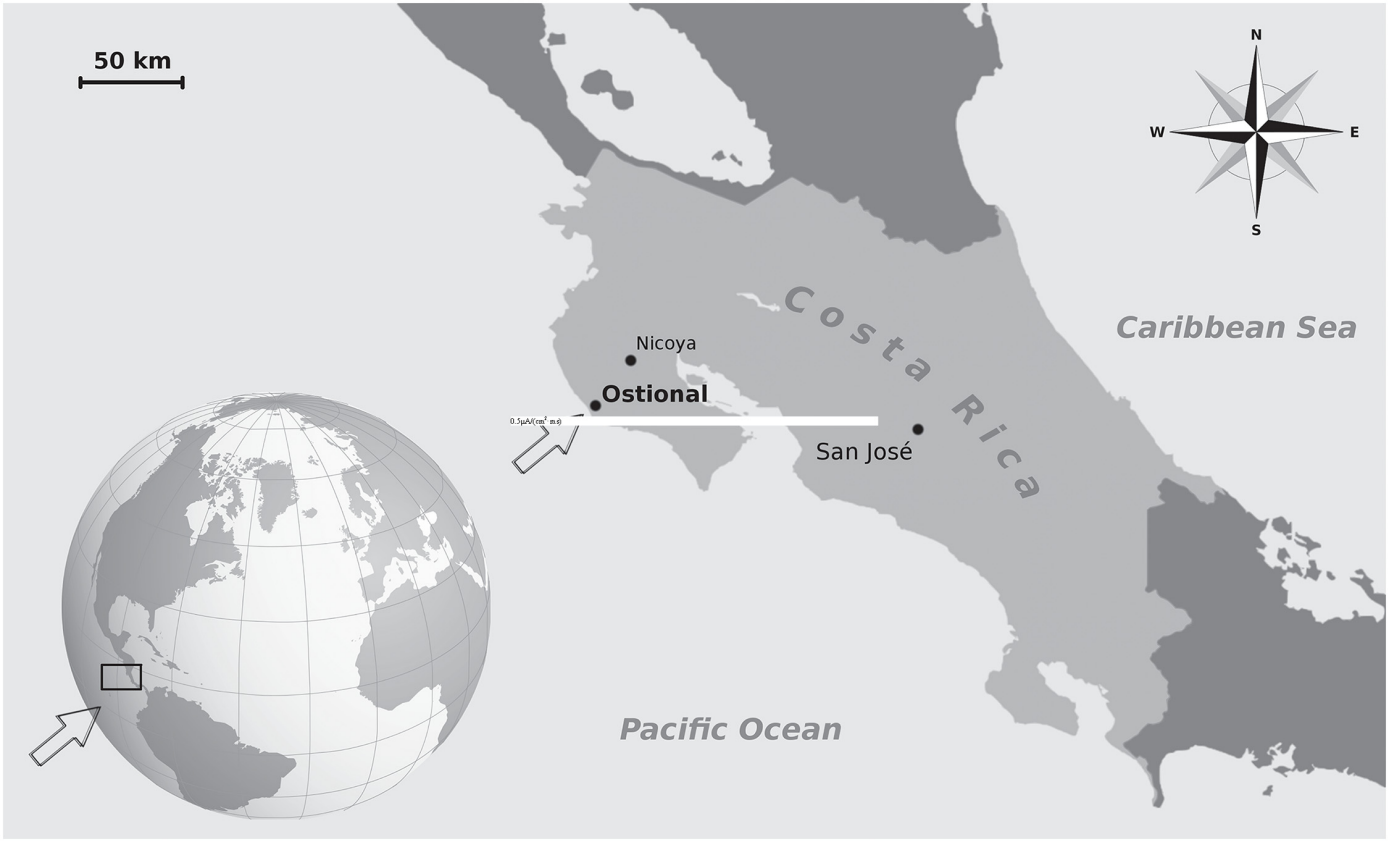
Sampling area. Location of the sampling beach where olive ridley turtles (*Lepidochelys olivacea*) lay their eggs during *arribada* events. The map is a representation based on openstreetmap cartography distributed under CC BY-SA 2.0 license (http://creativecommons.org/licenses/by-sa/2.0/). Part of this work is also based on "brosen windrose" image (http://commons.wikimedia.org/wiki/File:Brosen_windrose.svg#filelinks) distributed under CC BY 2.5 license.

Samples were collected during the first three days of olive ridley *arribada* in the second week of October 2013, from the principal nesting beach (approximately 800 m long) of Ostional on the Pacific coast of Costa Rica (Fig 1). Ostional, one of the largest *arribada* beaches in the world, lies within the Ostional Wildlife Refuge [10]. Before sampling, the curved carapace length notch-to-tip (CCL) of each turtle was measured to the nearest centimetre [11].

### Material collection and preparation for microscopic observation of diatoms

Carapaces of nesting olive ridleys were shaved with a razor blade and random samples (n > 200) of about 20 cm^2^ were taken. At least 3 samples were taken from each of the 55 female turtles sampled. Collected material was immediately placed in 50 ml plastic containers and preserved with 4% formaldehyde solution in sea water. Subsequently, the samples were treated in two different ways. For diatom counting and growth form analysis, ca. 1 cm^2^ subsamples were cut from each of the carapace pieces collected. Sections were then dehydrated through a 25, 50, 60, 70, 80, 90, 95, 100% alcohol series, treated with a Critical Point Dryer (K850 EMITECH), placed on aluminium stubs and sputter-coated with platinum using a DESK V HP TSC Cold Sputter Coater. For taxonomic examination, small pieces (ca. 2 cm^2^) of collected material were digested with boiling concentrated acid (64% nitric acid and 97% sulphuric acid added at a 1:3 volume ratio respectively), rinsed abundantly with distilled water, centrifuged and decanted. Prior to SEM observations, clean material was mounted on aluminium stubs and sputter-coated with platinum.

### Microscopic observations and diatom counts

As preliminary observations using a light microscope indicated that diatom communities associated with different turtle specimens were structurally highly similar, samples from 38 turtles were selected for further detailed observation and analyses under a Zeiss Supra 40 SEM. Diatoms were identified and enumerated on a surface area of ca. 2 mm^2^ of each of the 3 subsamples derived from the 38 turtles at magnifications ranging between 400x to 60000x. Community quality indicators including Margalef’s species-richness d (d = (S—1)/(log_e_N), where S = number of species, N = number of individuals), Shannon-Wiener diversity H’ (log_e_), and Pielou’s evenness index J’ (J’ = H’ /log_e_S) were calculated for each of the samples. Statistical analyses were performed using PRIMER Ver. 6 [12] software.

### Macroscopic epibionts

Macro-epibiont samples were collected from the external surfaces of olive ridley turtles during nesting. They were removed using a pocket-knife or tweezers, and the location from which they were taken was recorded. Samples for morphological identification were preserved in 70% ethanol and the prevalence (i.e. the percentage of turtles upon which a single epibiont species was found) was calculated. Representative specimens are deposited in the Zoological Collection of the University of Costa Rica.

## Results

**Fig 2:**
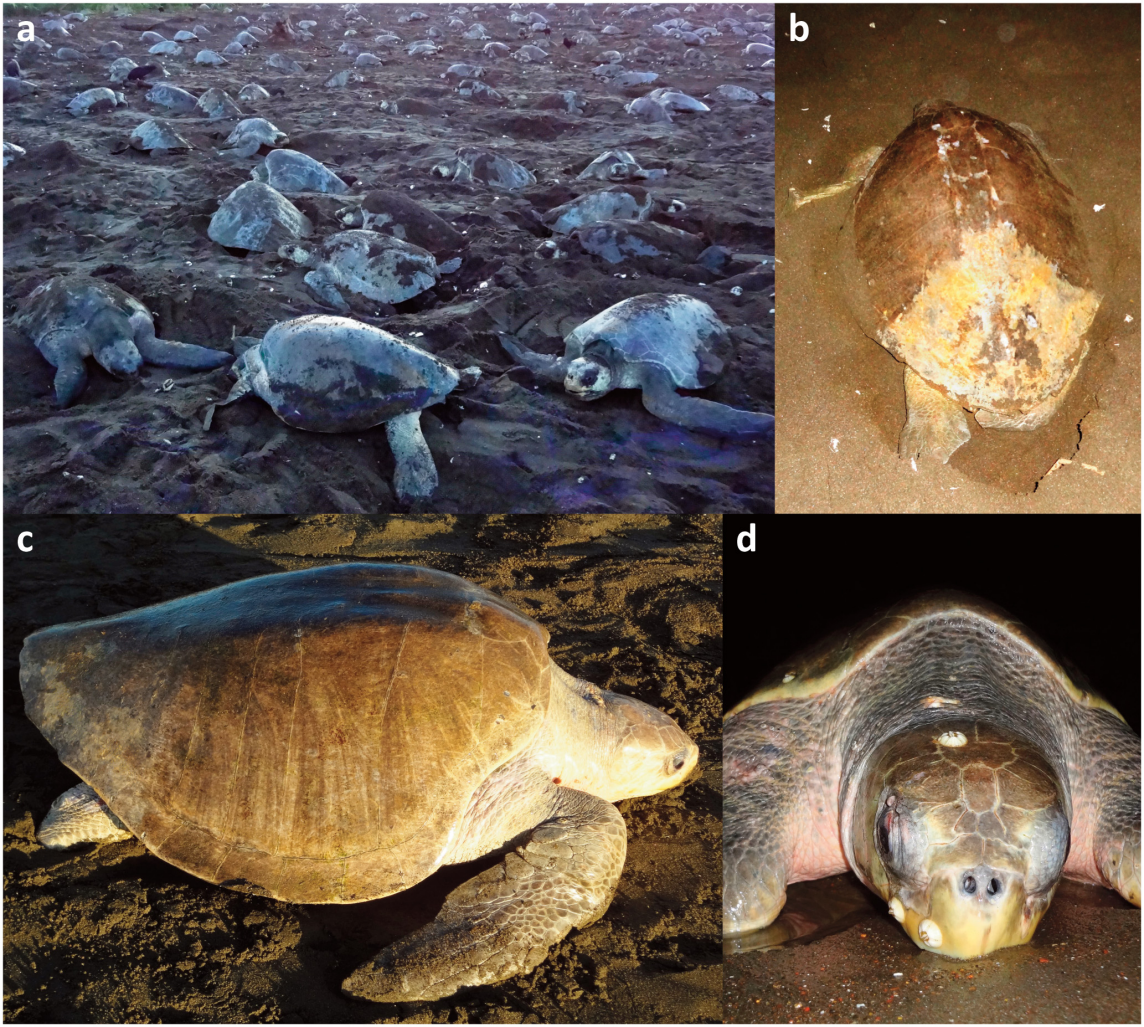
Olive ridley during the mass nesting event (arribada) at Ostional beach, Costa Rica in October 2014. a) Females emerged from the ocean after congregating offshore. b & c) Nesting turtle. Brownish biofilm covers the entire carapace but macroepibionts (e.g. macroalgae) prevail on its posterior parts. d) Olive ridley and epizoic barnacles.

Curved Carapace Length of the 55 examined nesting females ranged from 65 to 71 cm. Eleven species of macro epibionts were present on 2 to 85.5% (*Stomatolepas elegans*) of the examined turtle individuals (Table 1). Neither epizoic macroalgae nor sessile fauna covered the turtle carapace in a uniform manner. Filamentous algae were concentrated on the posterior and lateral parts of the carapace, while barnacles presented an irregular distribution (Fig 2). A microbial biofilm, however, could clearly be seen to cover the entire carapace.

**Table 1:** Epibiotic macro fauna found on 55 nesting olive ridley turtles (*Lepidochelys olivacea*) from Ostional, Pacific coast of Costa Rica.

Taxa	Prevalence (%)	Anatomical site
Arthropoda: Cirripedia		
*Conchoderma auritum* (L.)	5.5	Carapace
*C*. *virgatum* Spengler	3.6	Carapace
*Lepas hilli* Leach	9	Carapace
*Platylepas decorata* Darwin	2	Skin
*P*. *hexastylos* Fabricius	2	Skin
*Chelonibia testudinaria* (L.)	78	Carapace, head, skin
*Stomatolepas elegans* (Costa)	85.5	Skin
Arthropoda: Malacostraca		
*Podocerus chelonophilus* Chevreux & Guerne	3.6	Carapace
*Planes* sp.	2	Carapace
Anellida: Hirudinea		
*Ozobranchus branchiatus* Menzies	56.3	Skin
*O*. *margoi* Apàthy	2	Skin

**Fig 3:**
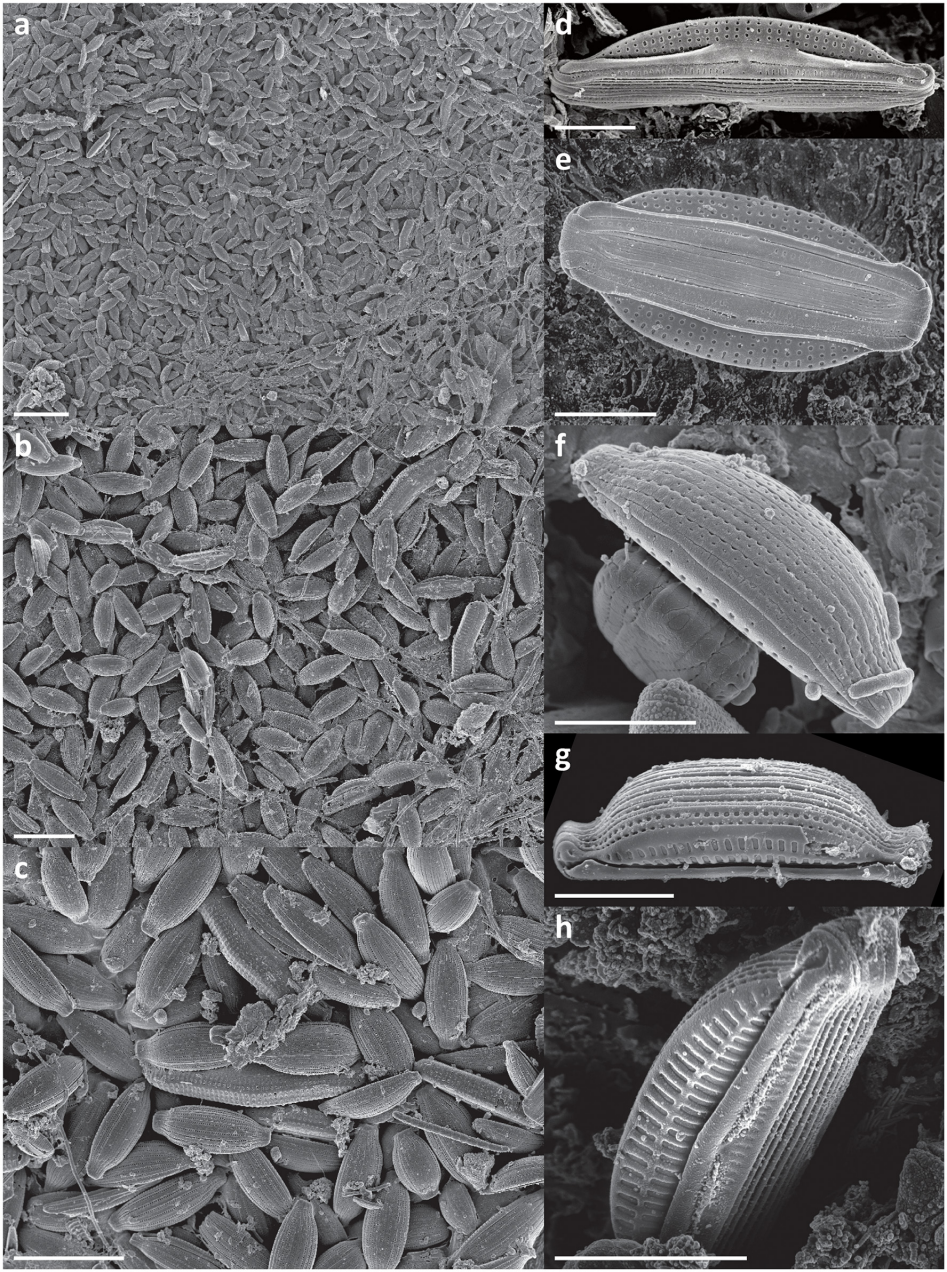
Scanning electron micrographs of epizoic diatoms associated with olive ridley carapace. a, b & c) *Amphora* spp. assemblage. d) *Amphora* sp. 1, side view. e) *Amphora* sp. 1, ventral view. f) *Amphora* sp. 1, dorsal view. g& h) *Amphora* sp. 2. Scale bars: a = 50 μm, b & c = 20 μm, d-h = 5 μm.

**Fig 4:**
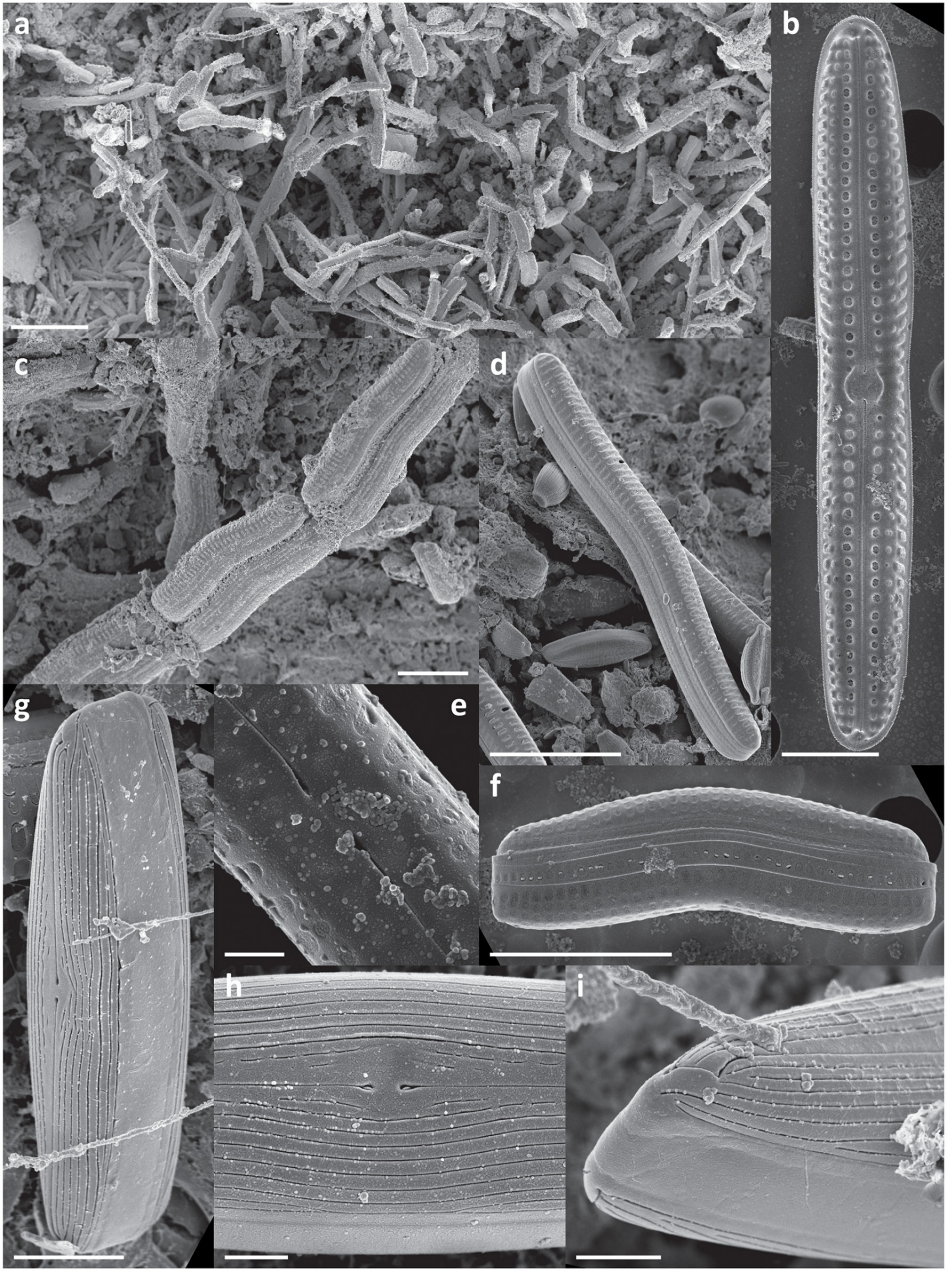
Scanning electron micrographs of epizoic diatoms associated with olive ridley carapace. a-e) *Achnanthes* cf. *pseudogroenlandica* var. *phinneyi*: a) monospecific cluster of A. cf. *pseudogroenlandica* var. *phinneyi* growing on the turtle carapace, b) internal view of the raphe valve, c) chain of the attached cells, d) single cell, external view, e) details of the central are, f) *Achnanthes* sp. 1, a single cell in girdle view. g-i) *Haslea* sp. 1: g) entire cell in external view, h) details of the central area, i) details of the raphe endings. Scale bars: a = 50 μm, b & g = 5 μm, c, d & f = 10 μm, e, h & I = 1 μm.

**Fig 5:**
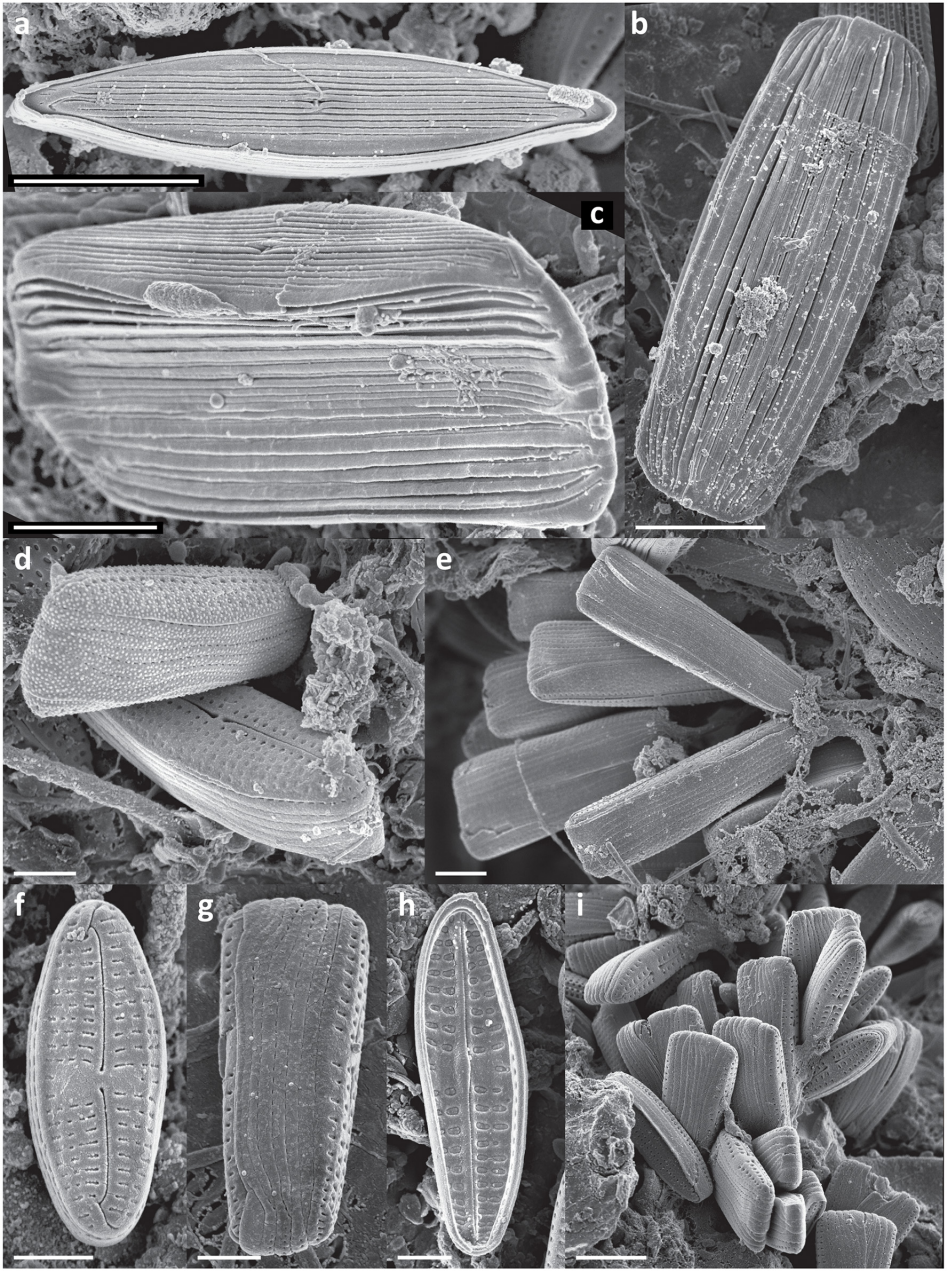
Scanning electron micrographs of epizoic diatoms associated with olive ridley carapace. a-c) *Proschkinia* sp.: a) valve view, b) diagonal view, c) girdle view. d & e) *Tripterion* sp. 1. f-i) *Tripterion* sp. 2: f) valve view (external), g) girdle view, h) valve view (internal), i) cluster of cells attached to the turtle carapace. Scale bars: a-c &i = 5 μm, d-h = 1 μm.

**Fig 6:**
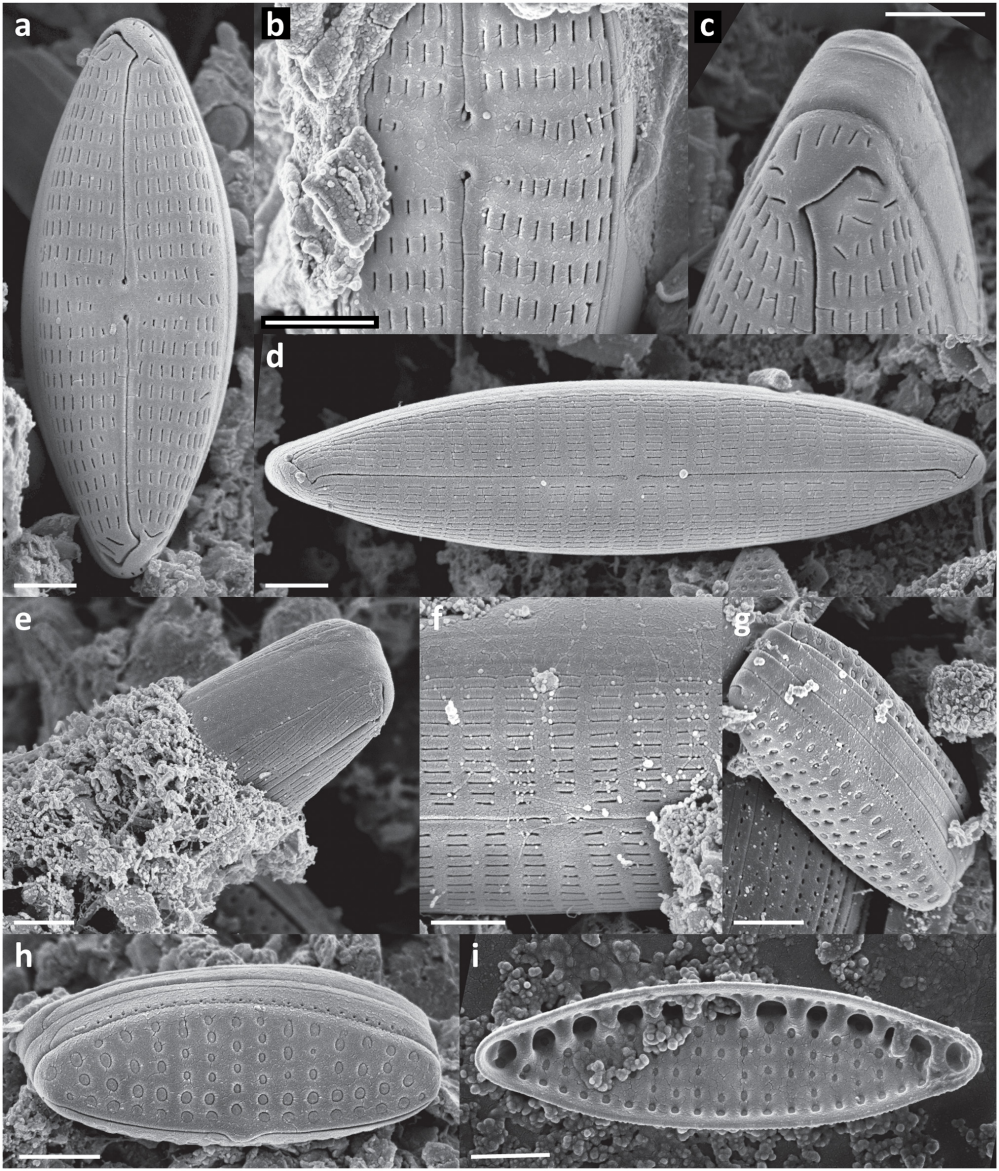
Scanning electron micrographs of epizoic diatoms associated with olive ridley carapace. a-c) *Navicula* cf. *rusticensis*: a) valve view, b) details of the central area, c) details of external raphe ending.d-f) *Navicula* sp. 1: d) valve view, e) cell immersed in the mucilage tube, f) details of the central area. g-i) *Nitzschia* sp. 1: g) diagonal view, h) valve view (external), i) valve view (internal). Scale bars = 1 μm.

**Fig 7:**
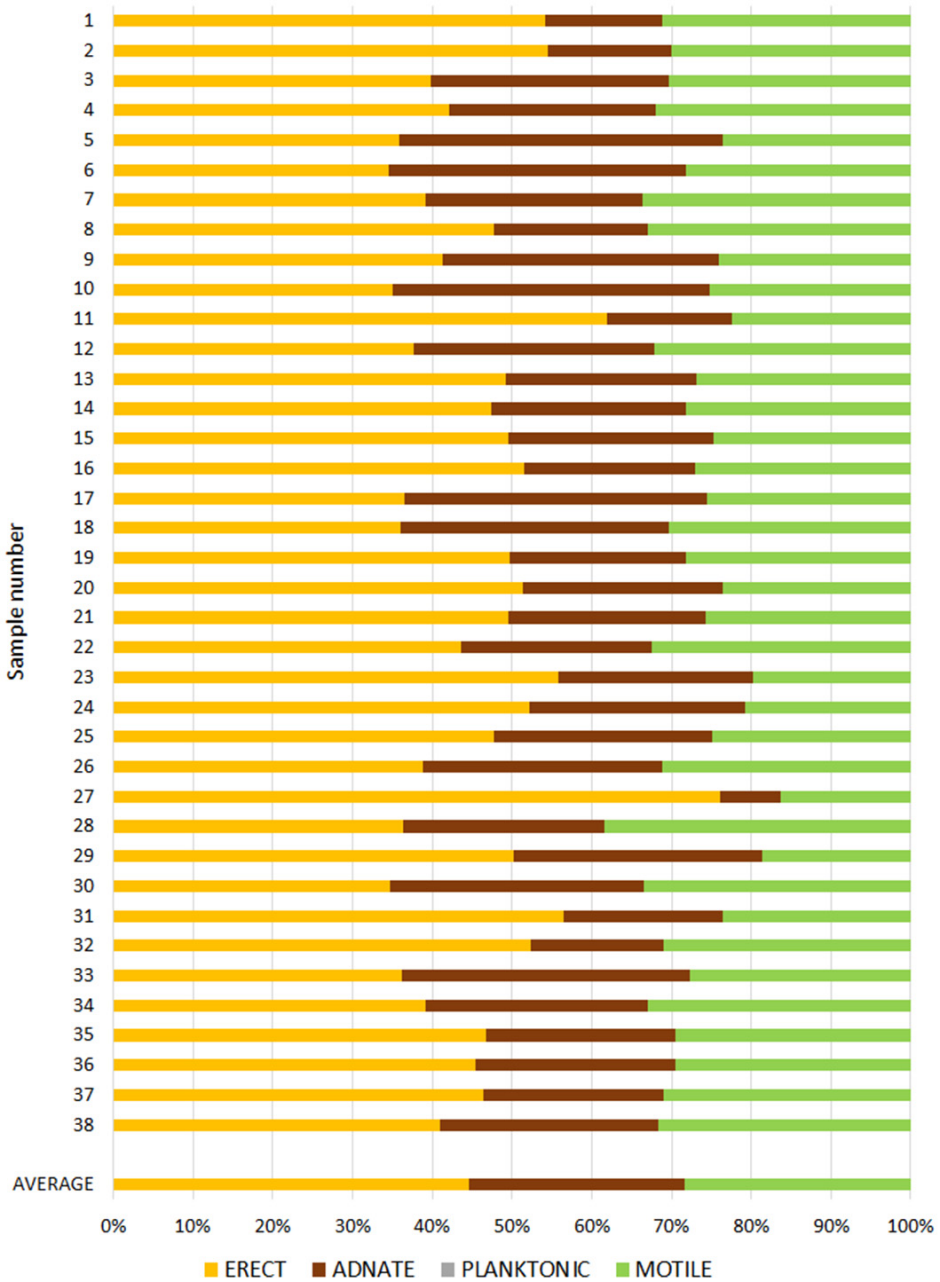
Diatom growth form percent contribution to total diatom abundance.

In every sample a complex, highly structured diatom community was observed. Twenty-one diatom taxa were found within the epibionts growing on the turtle carapaces (Table 2). Amongst these, eleven taxa (*Achnanthes* cf. *groenlandica*, *Achnanthes* cf. *pseudogroenlandica var*. *phinneyi*, *Achnanthes* sp., *Amphora* sp. 1, *Haslea* sp., *Navicula* cf. *rusticensis*, *Navicula* sp., *Nitzschia* cf. *inconspicua*, *Proschkinia* sp., *Tripterion* sp. 1, and *Tripterion* sp. 2; Figs 3, 4, 5 and 6) contributed more than 1% of the total diatom number on the surface of at least one turtle specimen examined. These 11 taxa occurred in at least 97% of the samples, while the other 10 species were present only occasionally (3–8% of the samples). Only seven species (*Achnanthes* cf. *groenlandica*, *Achnanthes* cf. *pseudogroenlandica var*. *phinneyi*, *Achnanthes* sp., *Amphora* sp. 1, *Nitzschia* cf. *inconspicua*, *Tripterion* sp. 1, and *Tripterion* sp. 2) contributed more than 3% of the total diatom number, all occurring in every sample examined (Table 2), and accounting for 91.5–99.3% of the total diatoms. Apart from rarely observed *Azpeitia nodulifera* (8% of samples; < 1% of the total diatom number), the diatom community was restricted to pennate diatoms. In general, communities associated with different turtle specimens were composed of the same diatom taxa, but in some cases the contribution of a single taxon to the total diatom community varied considerably. On average, *Tripterion* spp. and *Amphora* spp. were the most important components contributing 32.1 and 25.1% of total diatom number, respectively. The relative contribution of *Achnanthes* spp. varied the most, ranging from 1.5 to 62.6%, with a mean value of 15.3% (S1 Fig). In terms of diatom growth form, erect diatoms (*Achnanthes* spp., *Tripterion* spp.) dominated (25.7–81.7%, mean 47.5%), followed by motile (*Haslea* sp., *Navicula* spp., *Nitzschia* spp. *Proschkinia* sp.; 13–38.6%, mean 27.4%) and adnate forms (*Amphora* spp.; 3–58.2%, mean 25.1%; Fig 7, S2 Fig).

**Table 2:** Diatom taxa.

Taxa	Total abundance (%)	Prevalence (%)
*Achnanthes* cf. *groenlandica* Cleve & Grunow	1.5–42.6	100
*Achnanthes* cf. *pseudogroenlandica var*. *phinneyi* McIntire et Reimer	1.4–38.1	100
*Achnanthes* sp.	1.2–18.9	100
*Amphora* sp. 1	3–57.3	100
*Amphora* sp. 2	<1	13
*Azpeitia nodulifera* (Schmidt) Fryxell & Sims	<1	8
*Cocconeis neothumensis* var. *marina* De Stefano, Marino & Mazzella	<1	3
*Diploneis* litoralis (Donkin) Cleve	<1	3
*Grammatophora marina* (Lyngbye) Kützing	<1	3
*Haslea* sp.	<1–2.8	100
*Navicula rusticensis* Lobban	<1–3	100
*Navicula* sp.	<1–1.4	97
*Nitzschia* cf. *inconspicua* Grunow	9.6–34.4	100
*Nitzschia panduriformis* W.Gregory	<1	3
*Pinnularia quadratarea* (A.Schmidt) Cleve	<1	5
*Planothidium* cf. *delicatulum* (Kützing) Round & Bukhtiyarova	<1	3
*Pleurosigma* cf. *angulatum* (Queckett) W.Smith	<1	3
*Proschkinia* sp.	<1–2.8	100
*Trachyneis aspera* (Ehrenberg) Cleve	<1	5
*Tripterion* sp. 1	6.1–23.8	100
*Tripterion* sp. 2	12–32.3	100

Diatom taxa found on nesting olive ridley turtles (*Lepidochelys olivacea*) from Ostional with total abundance (%) and prevalence of occurrence (%) for each specific diatom taxa.

Although different diatom species dominated locally creating monospecific clusters, no bare areas were observed. Diatoms covered all available surfaces, ranging from 8179 ± 750 to 27685 ± 4885 cells mm^-2^ (17648 ± 5949 cells mm^-2^ on average) (Table 3). Values of the Margalef’s species-richness (d), Pielou’s evenness (J’), and Shannon-Wiener diversity index (H’) ranged from 0.87 to 1.38 (mean 1.08), 0.56–0.86 (mean 0.71), and from 1.41 to 2.1 (mean 1.73), respectively (Table 3). Many diatom cells were partly immersed or entirely encapsulated within an exopolymeric coat formed most likely by either the diatoms themselves or fungal or bacterial populations coexisting with the stratified diatom community (Figs 3, 4, 5 and 6).

**Table 3:** Diatom indices.

Sample	S	d	J'	H’	Total diatom abundance (cells mm^-2^) ± SD
1	12	1.07	0.61	1.51	12782 ± 1399
2	11	1.05	0.83	2	13086 ± 1257
3	12	1.09	0.62	1.55	24991 ± 4611
4	13	1.22	0.59	1.51	27685 ± 4885
5	11	0.99	0.61	1.46	19344 ± 846
6	14	1.3	0.64	1.7	17292 ± 1298
7	11	1.01	0.67	1.6	13807 ± 1549
8	13	1.28	0.56	1.44	15688 ± 1170
9	11	1.02	0.64	1.53	13539 ± 2961
10	11	1.05	0.76	1.81	15311 ± 1716
11	11	1	0.62	1.48	13761 ± 2287
12	12	1.1	0.57	1.41	24132 ± 2313
13	12	1.1	0.65	1.6	17965 ± 2342
14	12	1.2	0.81	2	14731 ± 2307
15	11	1.07	0.66	1.58	21743 ± 5304
16	11	1.08	0.79	1.88	16796 ± 3471
17	11	1.05	0.86	2.1	21754 ± 2506
18	11	1.1	0.86	2.1	22122 ± 2799
19	11	1.05	0.77	1.86	16534 ± 1533
20	11	1.01	0.72	1.72	16411 ± 4478
21	11	1.03	0.86	2.07	15408 ± 328
22	11	1.03	0.80	1.93	20925 ± 2428
23	12	1.22	0.81	2.01	14735 ± 1838
24	11	1.04	0.84	2.01	14853 ± 3789
25	11	1.04	0.84	2.02	17645 ± 5067
26	11	1.04	0.70	1.68	24422 ± 3807
27	11	1.03	0.68	1.64	8179 ± 750
28	14	1.38	0.62	1.64	14885 ± 967
29	11	1.02	0.69	1.67	14022 ± 1644
30	11	1.03	0.70	1.69	20661 ± 5353
31	11	1.04	0.72	1.73	13874 ± 830
32	13	1.25	0.68	1.74	15830 ± 1998
33	12	1.05	0.70	1.74	17648 ± 2163
34	11	1.01	0.77	1.84	22584 ± 4572
35	11	1.01	0.68	1.63	17664 ± 723
36	12	1.12	0.74	1.84	19789 ± 1291
37	10	0.87	0.67	1.55	17623 ± 910
38	11	1.02	0.66	1.57	20436 ± 1538
AVERAGE	11.5	1.08	0.71	1.73	17648 ± 5949

Number of diatom taxa found (S), values of the Margalef’s species-richness index (d), Pielou’s evenness index (J’), and Shannon-Wiener diversity index (H’) calculated for each turtle examined, and the total diatom abundance values with standard deviation (SD).

According to SIMPER (Similarity Percentages—species contributions) analysis, samples collected from each turtle contained high within-group average similarities (74.2–96%; on average 86.6%) and low inter-group dissimilarities (4.5–33.5%; on average 14.4%). ANOSIM (Analysis of Similarities) confirmed that diatom community associated with each turtle was not significantly different from the others in terms of both species composition (Global R = 0.095, p<0.05) and growth form structure (Global R = 0.075, p<0.05; S2 Fig).

## Discussion

Exploration of as yet unknown, undescribed marine habitats often yields new diatom taxa. Amongst these, some of the most intriguing forms are epizoic diatoms, which often have traits of obligate epibionts [13, 14, 15]. The nature of the close relationships between substrate organism and its epibionts requires further detailed investigation. However, some of the reported symbiosis-like associations [14, 16, 17] may in fact be species-specific and we may expect many new epizoic taxa to be discovered along with the examinations of generally understudied epizoon. In the present study, in spite of extensive literature research and consultation with diatom taxonomic experts, we found that the morphology of many of the observed taxa did not correspond precisely with descriptions of known diatom species. For the most common of these taxa SEM micrographs are provided (Figs 3, 4, 5 and 6). A detailed taxonomic analysis was not the specific objective of this survey but is clearly required in future.

The high density and specific composition of epizoic diatom communities may perform an ecological function through providing an unique microhabitat for benthic micro- and mesofauna in the otherwise rather uniform and inaccessible pelagic environment. Epizoic habitats, such as those provided by sea turtles, constitute patches of high benthic primary production and contribute to the biodiversity and productivity of the marine ecosystem. The magnitude of this contribution, however, remains unknown. The observed epibiotic diatom densities, reaching 27685 ± 4885 cells mm^-2^, are amongst the highest reported to date from various biotic substrates collected in different parts of the world [18, 19, 20].

Taking into consideration the sea turtle life cycle and habits (e.g. deep dives, periodical emerging to the water surface, long migrations), the turtle carapace may appear to be a stressful habitat for diatoms. Nevertheless, the associated diatom communities were well-developed and highly-structured. The high contribution of erect diatoms to the total diatom number indicates 3-dimensionality and vertical stratification. The fact that all examined olive ridley females supported well-developed diatom communities is in accordance with the Caine’s [21] observation that the mating activities in sea turtles probably do not significantly affect the epibiotic community, although further support for this assertion requires examination of the microepibionts present on both sexes of turtle. In the present study, copious amounts of rich organic film composed most likely of bacteria and diatom extracellular polymers were observed in every sample. Mucilage substances produced by diatoms in the form of various structures (pads, stalks, tubes, adhering films, etc.) have numerous functions and contribute greatly to their biological success. Extracellular polymeric substances (EPS), due to their specific morphology, ultrastructure and biochemistry, play an important ecological role, for example in protecting diatom cells from desiccation, harmful solar radiation or excessive grazing, and increasing the physical stability of the microbial community [22, 23, 24]. Moreover, according to some authors [25, 26, 27], the presence of a bacterial biofilm may promote the attachment of microalgae, including diatoms.

Epizoic diatom growth on sea turtle carapaces is likely to be a common phenomenon occurring in various marine habitats. The low species number and relatively stable species composition (low inter-sample dissimilarities) observed here may suggest a mutualistic relationship between the epibiont and the basibiont. While the potential benefits for the epizoic diatom are likely to include factors such as provision of an appropriate solid substrate as well as a potential nutrient and CO_2_ source [19, 28], any benefit to the sea turtle is not clear. It seems unlikely that microorganism such as diatoms, even when present in high densities, may significantly disadvantage the sea turtle. Nevertheless, by creating a specific microhabitat suitable for larger sessile and mobile organisms, diatoms may indirectly contribute to the negative effect of increased mass and friction. Alternatively, epibionts may provide camouflage or protection against desiccation. How important this may be for a sea turtle is open to conjecture. It is a remarkable fact, however, that the associated organisms influence many areas of the interactions between the host organism and its biotic and abiotic environment [29, 30].

It is known that many diatoms are sensitive to environmental parameter changes on very small scales [31]. This may underlie the observation that the rather unstable habitat provided by the sea turtle is occupied by few diatom species overall. These species have presumably adapted successfully to the unusual combination of conditions found on the turtle carapace, and are able to survive and develop dense populations there. While sessile diatoms depend strongly on hard substrates which allow them to complete their ontogenesis, the availability of solid surfaces often becomes a limiting factor [32, 33]. Any adaptation enabling a diatom to settle and develop on a living and moving substrate may therefore provide a competitive advantage. A specialised epibiont will benefit from reduced competition for space, as the living substrate may be poorly suited to other colonizers [29]. We speculate that the observed epizoic community in its present form may be a result of ecological vicariance: differences in the habitat of originally benthic species provided opportunities for specialization and speciation within local diatom groups [29, 34]. On the other hand, some of the taxa that usually occur together and are typical for a certain microhabitat may as well be common to the surrounding habitats [35]. According to some reports [36] mucilaginous attachments produced by several typically epibiotic diatoms allow the attachment of other nonmucilaginous cells. In this way, local unstalked diatoms may attach to the mucilaginous diatom substrate when a diatom-bearing sea turtle forages in the vicinity of the natural substrates of these diatoms. If so, there is potential for epizoic diatom communities to be used as biomarkers indicating foraging sites for different sea turtle populations. This novel approach might help to bridge the wide gap in general understanding of sea turtle foraging ecology and life cycle, and we encourage turtle researchers to consider the role of epizoic microorganisms (including diatoms) in their studies.

## Supporting Information

S1 Fig Dominant diatom taxa percent contribution to total diatom abundance. (TIF)

S2 Fig Diatom growth form contribution to total diatom abundance. Standard deviation bars indicate the inter-sample variability.
(TIF)
