# Supplementary figures and images for: Diatoms and Other Epibionts Associated with Olive Ridley (Lepidochelys olivacea) Sea Turtles from the Pacific Coast of Costa Rica
Source: PLoS One. 2015 Jun 17;10(6):e0130351. doi: 10.1371/journal.pone.0130351 (PMC4471233; doi:10.1371/journal.pone.0130351)

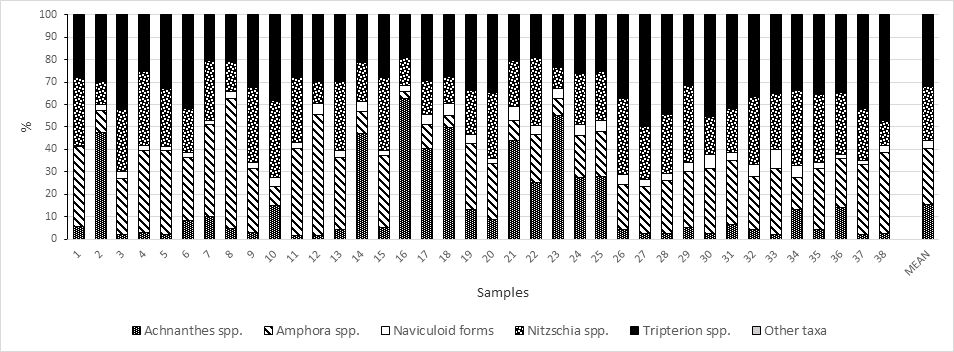

Supplement: S1 Fig — (TIF) [file pone.0130351.s001.tif]

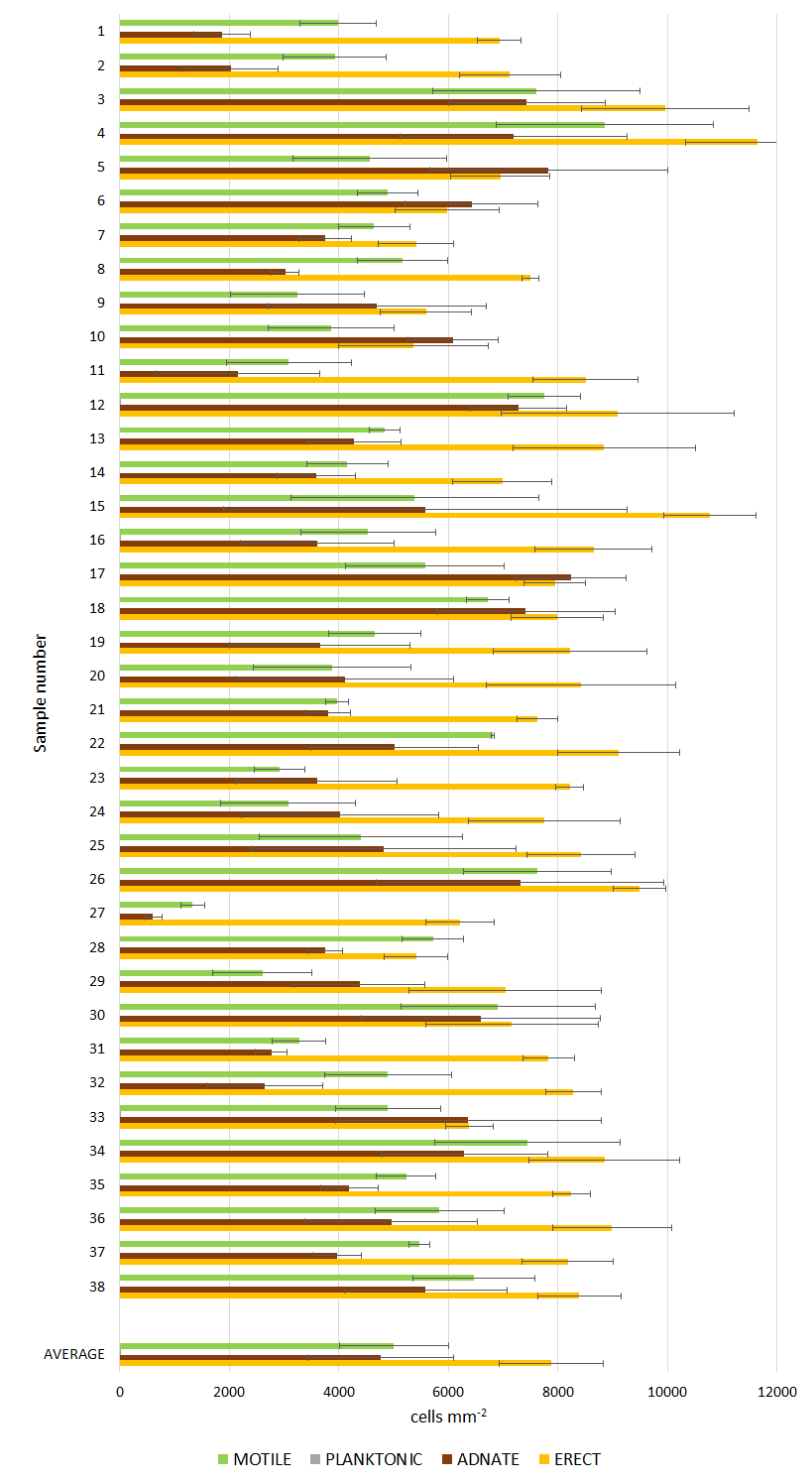

Supplement: S2 Fig — Standard deviation bars indicate the inter-sample variability. (TIF) [file pone.0130351.s002.tif]
